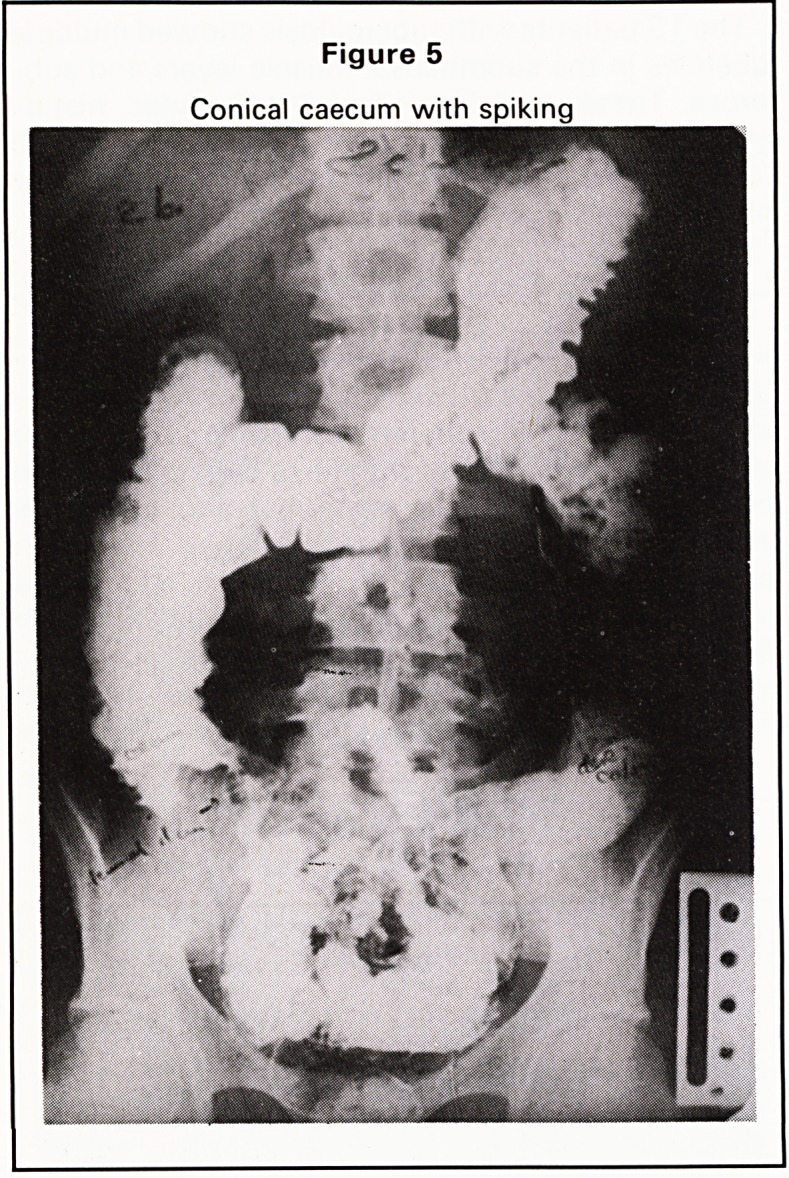# Chronic Granulomatous Cicatrising Enteritis—A Clinical Comparison of Crohn's Disease and Tuberculosis

**Published:** 1985-04

**Authors:** M. M. Guirgis

**Affiliations:** Professor of Surgery, Sohag, Egypt


### Chronic Granulomatous Cicatrising Enteritis

### A clinical comparison of Crohn's disease and tuberculosis

**M. M. Guirgis F.R.C.S. (Eng. and Ed.)**
Professor of Surgery, Sohag, Egypt

Chronic granulomatous cicatrising enteritis is defined as a condition causing a varying degree of stenosis and distortion of the bowel lumen with a fibroblastic reaction causing thickening of its wall. Our interest here lies in two conditions which so closely resemble each other that they create a diagnostic problem, Crohn's disease and tuberculosis. In spite of extensive clinical study and case reports from all over the world since Crohn described his cases in 1932,[1] the aetiology is still unknown. Intestinal tuberculosis while still common in Egypt is becoming increasingly rare in those parts of the world where tuberculosis is being brought under control. Difficulty in distinguishing these conditions on clinical, radiological and even histopathological grounds has suggested to Anand[2] that both are stages in one disease.

### MATERIALS AND RESULTS

An analysis of the clinical and pathological features of 25 cases has been made in an attempt to aid aetiological diagnosis. Twelve patients had Crohn's disease and 13 tuberculosis.

*Age incidence* – In this series ages ranged between 8 and 67 years in Crohn's disease with a maximum incidence in the third and fourth decades, while the tuberculous patients were between 15 and 55 with maximum incidence in the third decade.

*Sex incidence* – In Crohn's disease the sex incidence was equal being 6: while in tuberculosus there were 9 females to 4 males, these came from rural areas consuming a large amount of unpasteurised milk.

| *Presentation –* | Crohn | T.B. |
|---|---|---|
| 1. Long history of abdominal pain, diarrhoea and malaise | 3 | 0 |
| 2. Chronic intestinal obstruction, no abdominal mass | 2 | 4 |
| 3. Ditto with abdominal mass | 2 | 6 |
| 4. Mass in R.I.F. + persistent symptoms after appendicectomy | 3 | 0 |
| 5. Mass in R.I.F.? appendicular | 0 | 1 |
| 6. Faecal fistula+obstructive signs | 2 | 1 |
| 7. Signs of intussusception | 0 | 1 |
| | 12 | 13 |

From the above it seems that there are no specific diagnostic symptoms though malaise and diarrhoea or persistence of symptoms after appendicectomy may suggest Crohn's disease.

### LABORATORY INVESTIGATIONS

*Blood Picture* – In both conditions there was a slight degree of leucocytosis. The E.S.R. showed a marked rise in tuberculosis of 45–70 in the first hour while in Crohn's disease it was nearly normal.

*Tuberculin test* – It was negative in 7 patients with Crohn's disease and weakly positive in 5 while in tuberculosis it was positive in 6 patients, negative in 5 and unrecorded in 2. A positive result therefore favours a diagnosis of tuberculosis but a negative result does not rule it out.

### X-RAY FINDINGS

*Chest X-Ray* in the 25 patients showed a tuberculous lesion in only three of the 13 tuberculous patients.

*Barium studies –*

| *Ileum* | Crohn | T.B. |
|---|---|---|
| 1. Wide dilatation of small intestine (Figure 1) | 0 | 2 |
| 2. Ditto+flocculation suggesting malabsorption (Figure 2) | 0 | 1 |
| 3. Multiple narrowings with skipped lesions | 2 | 0 |

**Figure 1**

Wide dilatation of the small intestine

**Figure 2**

Dilatation of the small intestine showing flocculation of Barium, suggesting malabsorption

| | Crohn | TB |
|---|---|---|
| *Colon* | | |
| 1. Marked narrowing of caecum and ascending colon (Figure 3) | 0 | 1 |
| 2. Ascending colon narrowed with normal caecum | 0 | 1 |
| 3. Irregularity of outline with toothing suggesting ulceration (Figure 4) | 0 | 5 |
| 4. Caecum drawn up with widened ileocaecal angle | 0 | 2 |
| 5. Irregularity of caecum with narrowed angle. | 5 | 0 |
| 6. Conical caecum with spiking (Figure 5) | 3 | 0 |
| 7. 'Cobble stone' with skipped lesion | 0 | 1 |

In the ileo-caecal region the X-ray appearances are very similar. In the small intestine Crohn's disease produces longer strictures with less proximal dilatation than T.B. The short annular strictures of tuberculosis often produce marked proximal dilatation.

### OPERATIVE FINDINGS

Tuberculous enteritis took the form of multiple short strictures with severe stenosis and marked proximal dilatation while the lesions in Crohn's disease were long tubular segments with little diminution in calibre and minimal proximal dilatation. In the ileocaecal region there was marked thickening and oedema both of the gut and of the mesentery in both conditions.

Lymph gland involvement – In the three patients with tuberculous pulmonary lesions lymph gland involvement was not a prominent feature. In the other tuberculous patients lymph gland involvement was marked and in five patients there was naked eye caseation. In all the patients with Crohn's disease there was marked enlargement of the draining lymph glands but no caseation.

Some of the tuberculous patients showed subserous tubercles and free fluid. One patient had associated genital tract tuberculosis another patient had an intraluminal tuberculous mass causing an intussusception. In another patient a caseous mass produced a traction diverticulum in the lower ileum.

### HISTO-PATHOLOGICAL FEATURES

The 12 cases of Crohn's disease showed a more or less identical picture. Submucosal thickenings showed congestion and oedema with numerous granulomatous masses formed mainly of epithelioid cells and lymphocytes. Eosinophils and occasionally giant cells were seen in some sections. There was increased fibrosis in the submucosa and subserosa and in most cases also lymphangiectasis. A similar granulomatous infiltration was seen in the lymph nodes. There was no caseation.

The 13 patients with tuberculosis showed multiple tubercles in the submucosa, muscle layers and subserosa. These were formed of lymphocytes, histiocytes and foreign body giant cells mostly of the Langhans type. The lymph nodes were similarly infiltrated and there was extensive caseation.

### DIAGNOSIS

The presence of caseation is diagnostic of tuberculosis. In some tuberculous patients however caseation may be absent if there is high allergy with low resistance, in acute overwhelming infections and in chronic cases where caseation has been replaced by fibrosis and also in patients receiving a prolonged course of antituberculous therapy. The diagnosis is usually easy where the small bowel is involved but in the ileo-caecal cases it is more difficult and both conditions may exhibit the same

**Figure 3**

Marked narrowing of the caecum and ascending colon

**Figure 4**

Irregular outline of caecum with toothing suggesting ulceration

**Figure 5**

Conical caecum with spiking generic tissue response which may indeed be evoked by a wide variety of agents.

### REFERENCES

1. Crohn, B. B., Ginzburg, L. and Oppenheimer, G. B. (1932) *J.A.M.A.*, **99**, 1323.
2. Anand, S. S. (1956) *Anns. R.C.S. Eng.*, **19**, 205–222.

## Figures and Tables

**Figure 1 f1:**
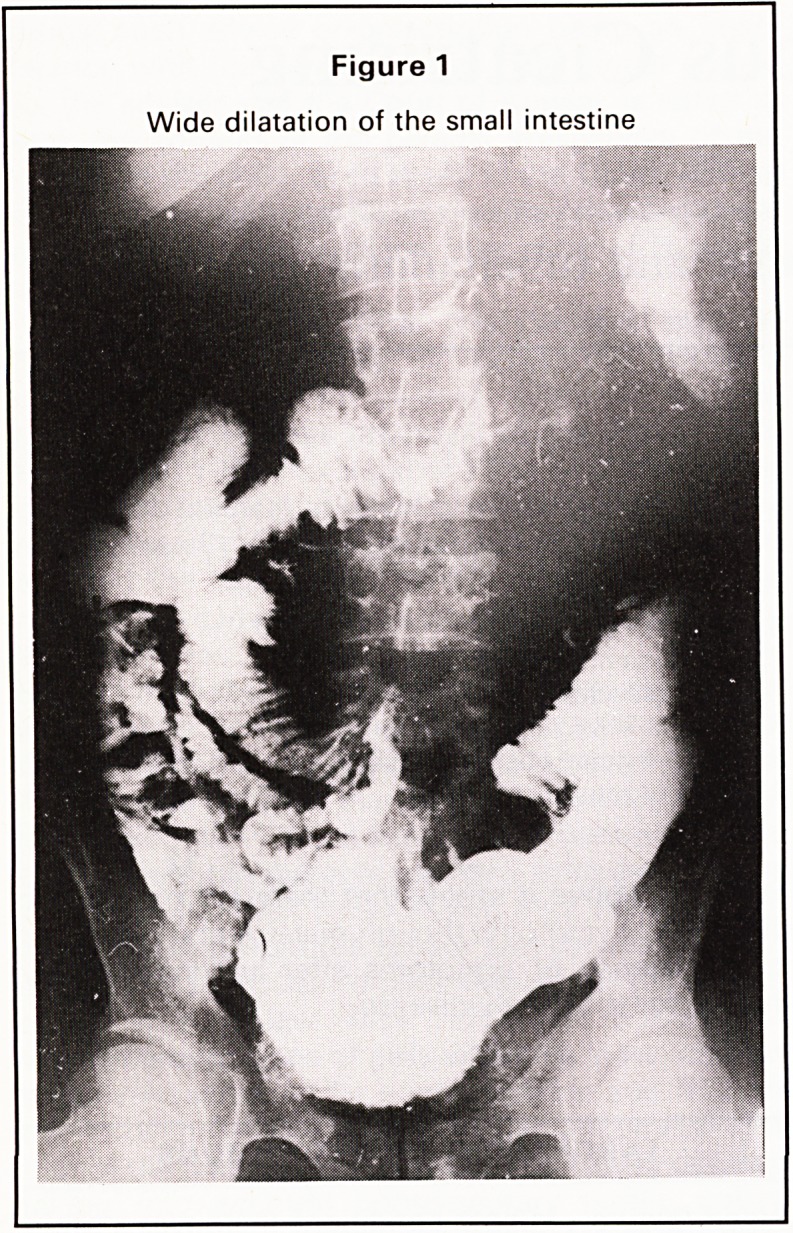


**Figure 2 f2:**
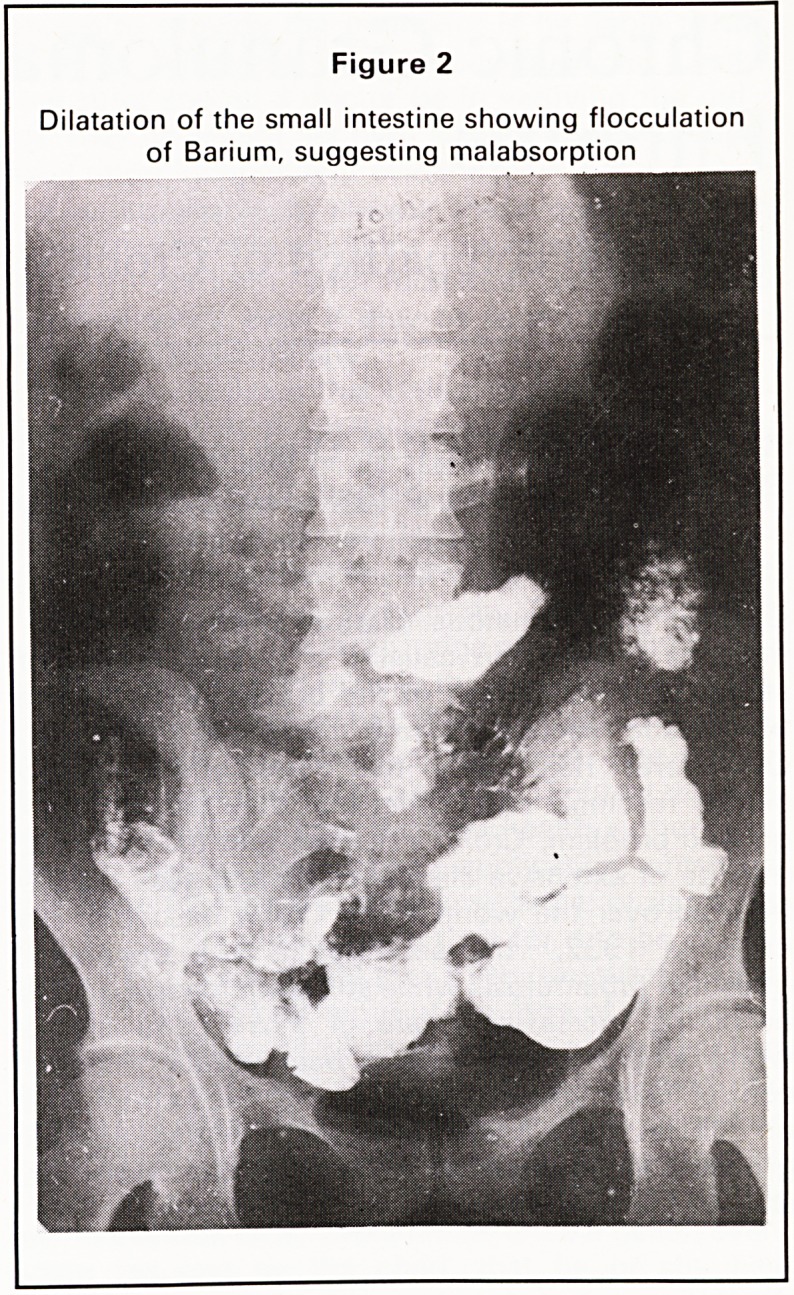


**Figure 3 f3:**
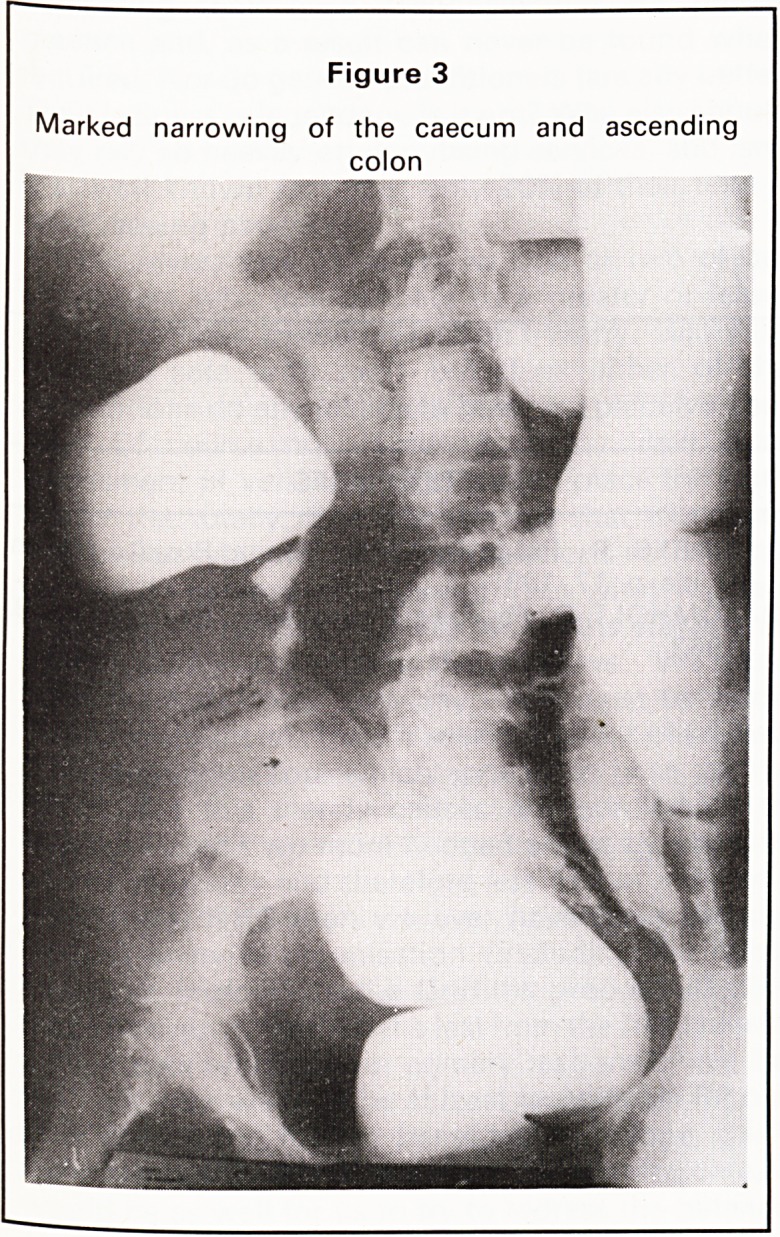


**Figure 4 f4:**
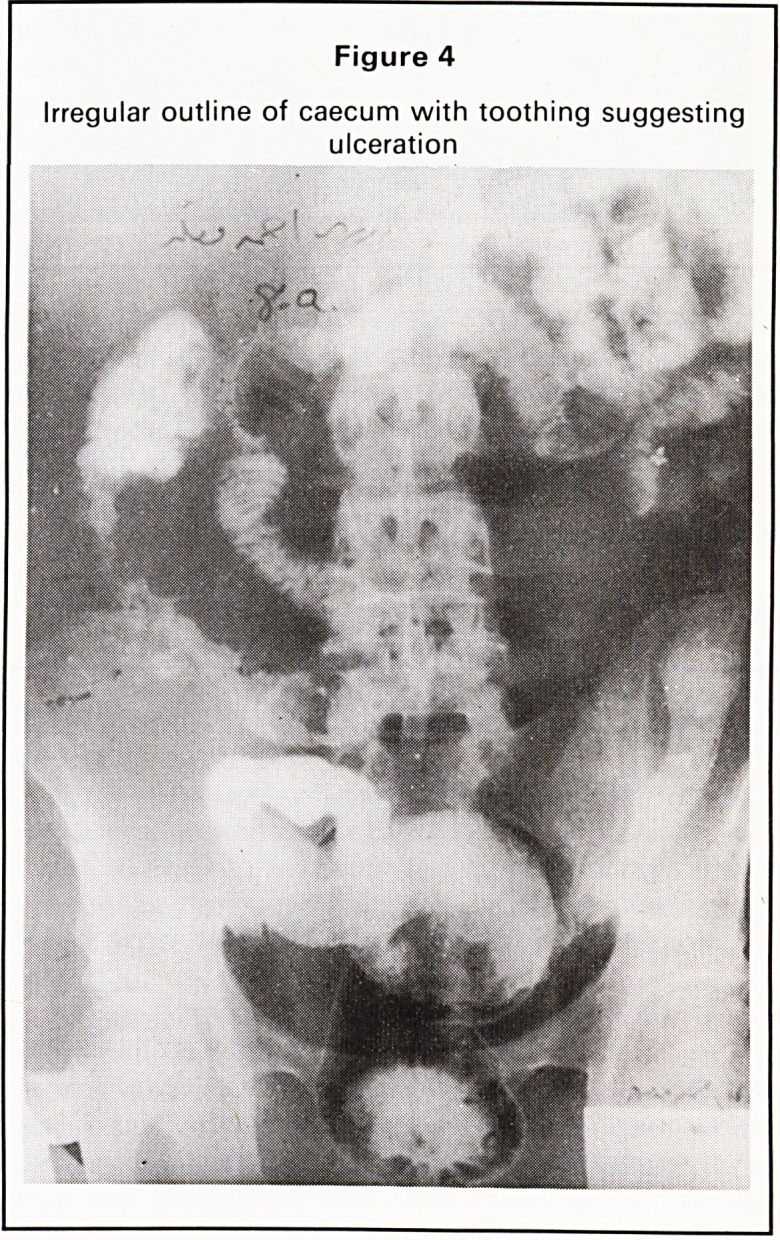


**Figure 5 f5:**